# A novel asymmetric synthesis of cinacalcet hydrochloride

**DOI:** 10.3762/bjoc.8.158

**Published:** 2012-08-24

**Authors:** Veera R Arava, Laxminarasimhulu Gorentla, Pramod K Dubey

**Affiliations:** 1 R&D Laboratory, Suven Life Sciences Ltd., Hyderabad, India; 2 Department of Chemistry, J. N. T. University, Hyderabad, India

**Keywords:** asymmetric synthesis, (*R*)-*tert*-butanesulfinamide, cinacalcet hydrochloride, naphthyl ethyl sulfinamide, regioselective *N*-alkylation

## Abstract

A novel route to asymmetric synthesis of cinacalcet hydrochloride by the application of (*R*)-*tert*-butanesulfinamide and regioselective *N*-alkylation of the naphthyl ethyl sulfinamide intermediate is described.

## Introduction

**Figure 1:**
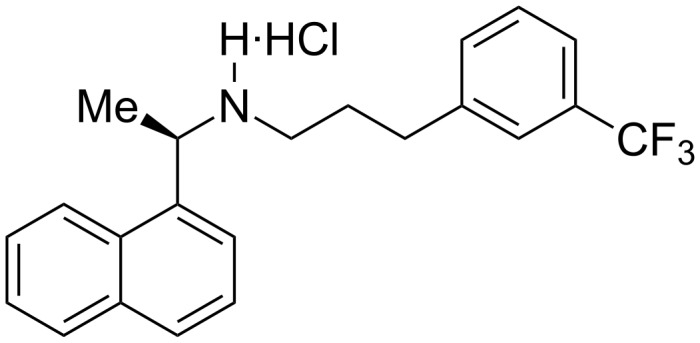
Cinalcet hydrochloride (CNC·HCl, **1**).

Cinacalcet hydrochloride (CNC·HCl, **1**, Figure 1) is the first active pharmaceutical ingredient (API) approved by the USFDA for the treatment of secondary hyperparathyroidism. It is sold under the trade names of Sensipar^®^ in USA and Mimpara^®^ in Europe. Hyperparathyroidism (HPT) is a condition characterized by the over-secretion of parathyroid hormone (PTH), a result of the failure of calcium receptors on parathyroid glands [1–2]. Calcimimetics are the agents that mimic the action of calcium to increase the sensitivity of these receptors to calcium, which inhibits the release of parathyroid hormone and lowers PTH levels in a very short time [3]. CNC·HCl (**1**) is the first and most successful drug among the calcimimetic agents, administered to patients with chronic kidney diseases.

## Results and Discussion

**Scheme 1:**
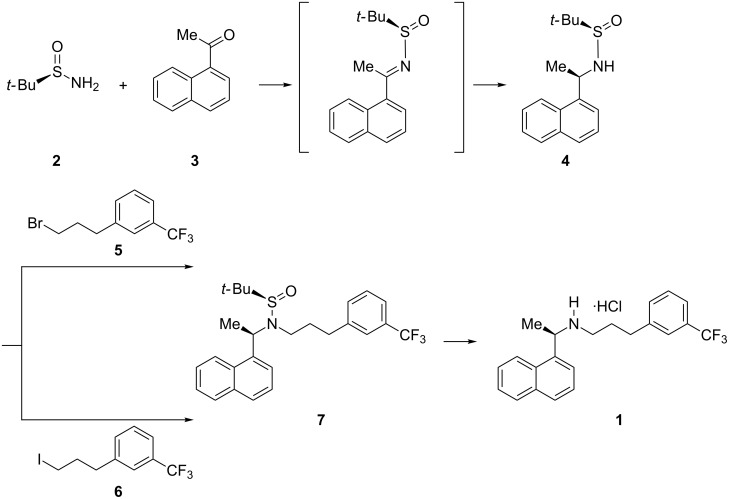
Asymmetric synthesis of **1**.

**Scheme 2:**
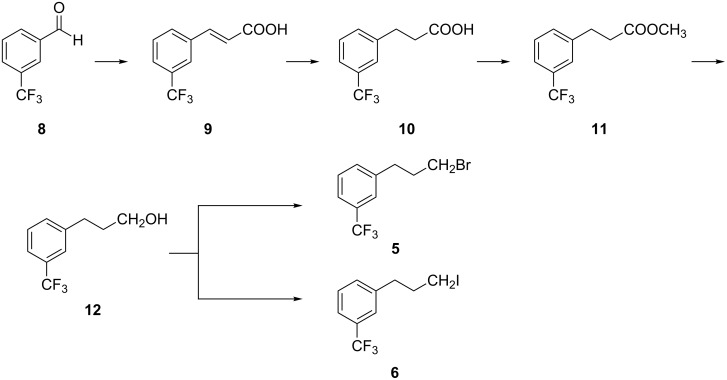
Synthesis of the intermediates **5** and **6** from **8**.

Several synthetic approaches have been reported for the synthesis of enantiopure CNC·HCl (**1**) [4–20]. In our quest to utilize the chiral *tert*-butanesulfinamides in asymmetric syntheses of chiral amine APIs in an industrial setting [21–23], we report a novel asymmetric synthesis of **1** (Scheme 1) based on (*R*)-*tert*-butanesulfinamide (**2**), which was developed and extensively studied by Ellman [24]. We have chosen 1-acetylnaphthalene (**3**) (Scheme 1) as a key starting material to produce the chiral amine center, and 3-trifluoromethylbenzaldehyde (**8**) as another key starting material for the preparation of the intermediates 1-(3-bromopropyl)-3-trifluoromethylbenzene (**5**) and 1-(3-iodopropyl)-3-trifluoromethylbenzene (**6**, Scheme 2).

**Scheme 3:**
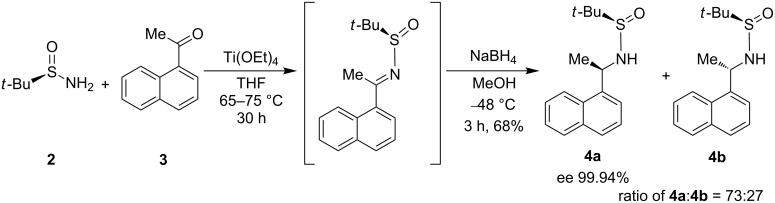
Asymmetric synthesis of naphthylethylsulfinamide **4**.

First, the enantiopure 2-methylpropane-2-sulfinic acid 1-(naphthalen-1-yl)ethylamide (**4**) was prepared by the condensation of **2** with **3** according to the earlier reported procedure [23]. The obtained crude is a diastereomeric mixture of **4a** and **4b** with a ratio of 73:27 (chiral HPLC analysis). From this crude mixture, **4a** was isolated in pure form by recrystallization from 10% ethyl acetate–hexanes in 68% yield with 99.94% ee (Scheme 3).

**Scheme 4:**
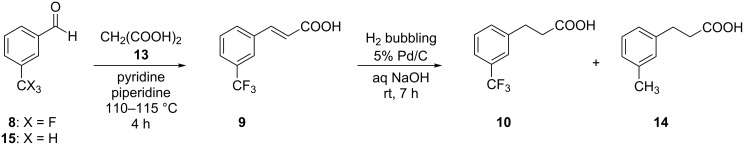
Conversion of **8** to **10**.

Malonic acid (**13**) was condensed with **8** and a catalytic amount of piperidine in pyridine under reflux to yield 3-(3-trifluoromethylphenyl)acrylic acid (**9**) in 90% yield [13]. The acid **9** was hydrogenated in the presence of 10% Pd/C catalyst in aqueous sodium hydroxide solution at ambient temperature, initially under 4.0 bar hydrogen overpressure to get 3-(3-trifluoromethylphenyl)propionic acid (**10**) in 98% yield. The desfluoro impurity **14** (around 8.2%, HPLC) was also detected (Scheme 4). Initially, **14** was reported in the literature [5] as a carryover impurity from bromo derivative **5** and from hydrogenation of **9** in methanol. The latter also lead to the reduction of the phenyl ring concomitant with double bond reduction. To reduce the formation of **14**, the reduction was tried to be carried out with 5% Pd/C and simply bubbling hydrogen gas into the reaction mixture at room temperature, and also by reducing the proportion of the catalyst (Table 1). The impurity formation was observed even at low catalyst loading. As a result, the impurity was removed by recrystallizing the crude product (Table 1, entry 5) in *n*-hexane at 0 °C twice and the pure product was obtained in 90% yield. The obtained product purity was >99% (GC–MS) which was sufficient for further conversions.

**Table 1:** Screening of reduction conditions (**9**→**10**).

entry	catalyst		H_2_ overpressure (bar)	time (h)	temperature (°C)	crude yield (%)^a^	**10** (%)	**14** (%)
	Pd/C (%)	(mol %)						

1	10	10	4.0	1	30–35	98	91.6^b^	8.2^b^
2	5	10	4.0	3	30–35	95	97.7^b^	2.2^b^
3	5	10	0^c^	5	30–35	96	87.3^d^	12.6^d^
4	5	5	0^c^	5	25–30	94	94.9^d^	5.1^d^
5	5	2	0^c^	7	25–30	95	98.8^d^	1.2^d^

^a^Isolated yield; ^b^% yield from HPLC; ^c^bubbling (1 atm); ^d^% yield in GC–MS.

We determined that **14** was not a carryover impurity from **8** by spiking analysis of **8** and **15** in gas chromatography. Both were clearly separated and **15** was absent in **8**. The impurity **14** was further structurally assigned by its synthetic preparation starting from **15**.

**Scheme 5:**
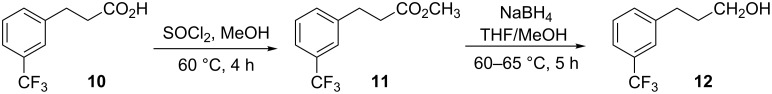
Synthesis of alcohol intermediate **12** from **10**.

The obtained propionic acid **10** was esterified to its methyl ester **11** with methanol and thionyl chloride under reflux for 4 h in 97% yield [13]. This ester **11** was reduced with NaBH_4_ in THF and methanol under reflux for 5 h [25] to afford 3-(3-trifluoromethylphenyl)propan-1-ol (**12**) in 95% yield (Scheme 5).

**Scheme 6:**
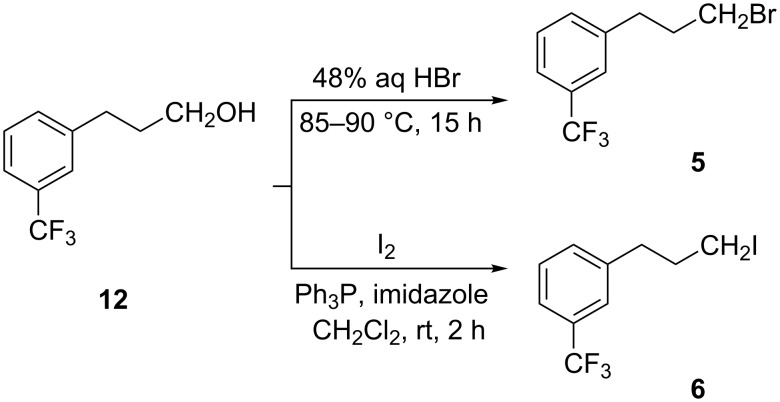
Synthesis of bromo **5** and iodo **6** derivatives.

From this alcohol **12**, both the bromo **5** and iodo **6** derivatives were prepared. Bromo derivative **5** was prepared simply by heating **12** in 48% aqueous HBr solution under reflux for 15 h. The obtained crude was purified by passing it through a silica gel plug with *n*-hexane to afford **5** in 82% yield. Iodide **6** was prepared by reacting **12** with molecular iodine in the presence of triphenylphosphine and imidazole in CH_2_Cl_2_ at room temperature for 2 h to give the product in 85% yield (Scheme 6).

**Scheme 7:**
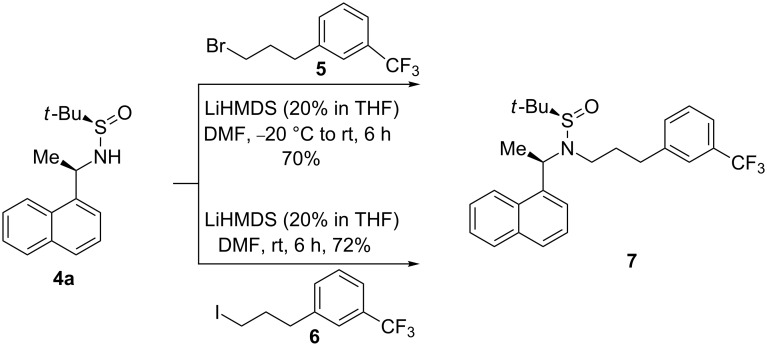
Regioselective *N*-alkylation of naphthyl ethyl sulfinamide **4a**.

**Scheme 8:**
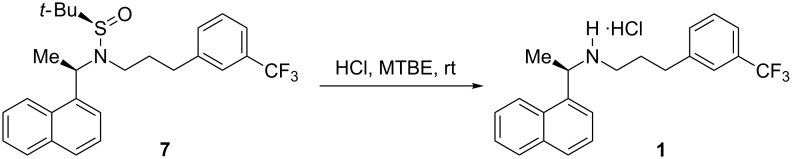
Acid hydrolysis of *N*-*tert*-butanesulfinyl group in **7**.

Regioselective *N*-alkylation of *N*-*tert*-butanesulfinamides was difficult to achieve as *S*-alkylation can also be possible [26]. To our knowledge, limited procedures were reported for the regioselective *N*-alkylation of *N*-*tert*-butanesulfinylamides [27–28]. Following the procedure reported in literature [26], initial experiments were conducted with **4** and **5**, and **4** and **6** in DMF with 2.0 equiv of LiHMDS at −20 °C to room temperature, which resulted in an isolated yield of 40 and 44% pure product, respectively. To force the reaction to completion and to improve the yield, the alkylation was carried out in various combinations and under various conditions (Table 2). Surprisingly, reactions without DMF and only in solvents like THF or DMSO were not initiated at all. Different combinations of bases and catalysts were also screened for the reaction progress, i.e., *n*-BuLi, LDA, Cs_2_CO_3_ with dppf/PdCl_2_, K_2_CO_3_ with Cu(I)/L-proline, NaH and TEA. All of them failed to initiate the reaction. Only LiHMDS worked well for the regioselective *N*-alkylation of sulfinylamide **4a** in a better yield (72% isolated pure product) than the earlier reported procedures for **1** (Scheme 7). Finally, hydrolysis of **7** dissolved in MTBE with conc. HCl at ambient temperature liberated the pure **1** (Scheme 8).

**Table 2:** Conditions for regioselective *N*-alkylation of naphthylethylsulfinamide **4a**.

entry	intermediate	base/solvent/catalyst	time (h)	temperature (°C)	**7** yield (%)^a^

1	**5**	LiHMDS (2.0 equiv)/DMF:THF	6	−20 to rt	41
2	**5**	LiHMDS (2.5 equiv)/DMF:THF	6	−20 to rt	44
3	**5**	LiHMDS (3.0 equiv)/DMF:THF	6	−20 to rt	50
4	**5**	LiHMDS (4.0 equiv)/DMF:THF	6	−20 to rt	66
5	**5**	LiHMDS (5.0 equiv)/DMF:THF	6	−20 to rt	69
6	**5**	LiHMDS (7.0 equiv)/DMF:THF	6	−20 to rt	70
7	**5**	LiHMDS (2.0 equiv)/THF	24	−20 to rt	NR
8	**5**	KO*t*-Bu (2.0 equiv)/DMF:THF	24	−20 to rt	10
9	**6**	LiHMDS (2.0 equiv)/DMF:THF	6	−20 to rt	44
10	**6**	LiHMDS (2.5 equiv)/DMF:THF	6	−20 to rt	48
11	**6**	LiHMDS (3.0 equiv)/DMF:THF	6	rt	55
12	**6**	LiHMDS (4.0 equiv)/DMF:THF	6	rt	70
13	**6**	LiHMDS (5.0 equiv)/DMF:THF	6	rt	71
14	**6**	LiHMDS (7.0 equiv)/DMF:THF	6	rt	72
15	**6**	LiHMDS (2.0 equiv)/THF	24	rt	NR
16	**5**	LiHMDS (4.0 equiv)/THF/Pd(dppf)Cl_2_	6	−20 to rt	NR
17	**5**	*n*-BuLi (1.1 equiv)/DMF:THF	24	−20 to rt	NR
18	**5**	LDA (2.0 equiv)/DMF:THF	24	−20 to rt	NR
19	**5**	Cs_2_CO_3_ (2.5 equiv)/DMSO/Pd(dppf)sCl_2_	24	rt	NR
20	**5**	K_2_CO_3_ (2.0 equiv)/NMP/CuI:L-proline	24	rt	NR
21	**5**	NaH (1.5 equiv)/THF	24	0 to 65	NR
22	**5**	TEA (2.0 equiv)/THF	24	rt	NR
23	**12**	THF/Raney-Ni	24	rt	NR

^a^Isolated yield (NR: no reaction).

## Conclusion

In summary, a novel stereoselective and short synthesis of (*R*)-cinacalcet hydrochloride by the application of (*R*)-*tert*-butanesulfinamide and regioselective *N*-alkylation of the naphthylethylsulfinamide intermediate was achieved in good yield.

## Experimental

Experiments were conducted under nitrogen atmosphere unless stated otherwise. All solvents and reagents were reagent grade pure and used without further purification. All melting points were determined on Polmon MP-96 melting point apparatus. ^1^H and ^13^C NMR spectra were recorded using a Bruker 400 MHz spectrometer (400 and 100 MHz, respectively) with TMS as internal standard. Mass spectra were recorded on a Perkin-Elmer mass spectrometer operating at an ionization potential of 70 eV. IR spectra were recorded on a Perkin-Elmer spectrophotometer as KBr pellets or neat. Analytical TLC is conducted on E-Merck 60 F_254_ aluminium-packed silica gel plates (0.2 mm). Developed plates were visualized under UV light or in an iodine chamber. Chiral HPLC analyses were recorded with on a Waters Alliance 2695 chromatograph with a 2487 UV detector.

**Preparation of 2-methylpropane-2-sulfinic acid (1-naphthalen-1-ylethyl)amide (4):** (*R*)-*tert*-Butanesulfinamide (**2**) (16.14 g, 0.133 mol) was added to a solution of titanium tetraethoxide (54.0 g, 0.236 mol) and 1-acetylnaphthalene (**3**, 20.0 g, 0.117 mol) in THF (200.0 mL) under an N_2_ atmosphere and the mixture was refluxed at 65–70 °C for 30 h. Upon completion, as determined by TLC, the reaction mixture was first cooled to rt and then to −48 to −52°C with a dry ice/acetone bath. NaBH_4_ (6.66 g, 0.176 mol) was added portion wise at −48 to −52 °C, and the mixture was stirred at −48 °C until the reduction was complete. Then, methanol (20.0 mL) was added drop wise until gas evolution stopped. The resulting mixture was poured into an equal volume of brine with rapid stirring. The resulting suspension was filtered through a pad of celite, and the bed was washed with ethyl acetate. The filtrate was extracted with ethyl acetate. The combined organic portions were dried over anhydrous Na_2_SO_4_, filtered, and concentrated to obtain the crude product. This crude product was crystallized from an *n*-hexane/ethyl acetate mixture (9:1 ratio) to get pure **4** as a pale yellow crystalline solid. The remaining product from the mother liquor was isolated from column chromatography with *n*-hexane/ethyl acetate (9:1). Yield = 22.0 g (68%); mp 97.0–98.2 °C; [α]_D_^23^ −116.8 (*c* 1.0, CHCl_3_); chiral purity (HPLC): 99.97% (*R*-isomer) and 0.03% (*S*-isomer); IR (KBr): 3220 (sharp, strong, –NH), 1057 (sharp, strong, –SO) cm^−1^; ^1^H NMR (CDCl_3_/TMS) δ 1.24 (s, 9H, C(CH_3_)_3_), 1.70 (d, *J* = 6.5, 3H, –CH_3_), 3.62 (s, 1H, –NH, D_2_O exchangeable), 5.39 (q, 1H, –CH), 7.46–7.62 (m, 4H, ArH), 7.81 (dd, *J_1_* = 29.0, *J_2_* = 8.0, 2H, ArH), 8.24 (d, *J* = 8.4, 1H, ArH); ^13^C NMR (CDCl_3_/TMS) δ 139.05, 133.88, 130.36, 128.89, 128.35, 126.40, 125.72, 123.42, 123.01, 55.40, 49.21, 22.55, 21.71; MS *m*/*z*: 276 [M + 1]^+^.

**Preparation of 2-methylpropane-2-sulfinic acid (1-naphthalen-1-ylethyl)-[3-(3-trifluoromethylphenyl)propyl]amide (7):** Amide **4** (3.0 g, 0.010 mol) was dissolved in DMF (9.0 mL) at rt under an N_2_ atmosphere. To the resulting solution, LiHMDS (63.85 mL, 0.070 mol) was added drop wise at rt over a period of 1 h. After stirring for 10 min, **6** (3.14 g, 0.010 mol) diluted with THF (3.0 mL) was added at rt over a period of 15 min, stirred for another 6 h at rt, and 15% ammonium chloride solution (30.0 mL) was added slowly. Then, the product was extracted with ethyl acetate. Evaporation of solvent and column chromatography of the crude product furnished **7** as a thick pale yellow syrup. Yield = 3.61 g (72%); chiral purity (HPLC): 99.9%; IR (neat): absence of 3220 (–NH), 2949, 2868, 1327, 1162, 1124, 1072, 798 and 779 cm^−1^; ^1^H NMR (CDCl_3_/TMS) δ 0.80 (s, 9H, C(CH_3_)_3_), 1.78 (d, *J* = 6.7, 3H, –CH_3_), 1.98 (m, 1H, –CH_2_), 2.28 (m, 1H, –CH_2_), 2.66 (m, 2H, –CH_2_), 2.90 (m, 1H, –CH_2_), 3.31 (m, 1H, –CH_2_), 5.29 (q, 1H, –CH), 7.22–7.53 (m, 6H, ArH), 7.60 (d, *J* = 7.0, 1H, ArH), 7.79 (d, *J* = 8.1, 1H, ArH), 7.86 (d, *J* = 7.7, 1H, ArH), 8.12 (d, *J* = 8.4, 1H, ArH); ^13^C NMR (CDCl_3_/TMS) δ 142.0, 137.16, 133.72, 131.75, 131.24, 130.81, 128.88, 128.77, 128.28, 126.10, 125.44, 125.22, 124.82, 123.02, 122.84, 57.27, 54.26, 43.13, 33.35, 30.84, 23.10, 18.47; MS *m*/*z*: 462 [M + 1]^+^.

**Preparation of 3-(3-trifluoromethylphenyl)acrylic acid (9):** Aldehyde **8** (50.0 g, 0.287 mol) was dissolved in pyridine (100.0 mL) and piperidine (0.5 mL) at rt. Malonic acid (**13**, 80.0 g, 0.768 mol) was added to this solution at rt and it was stirred for 15 min. Then, the reaction mixture was heated to 115–120 °C and stirred for another 4 h. After completion of the reaction, the reaction mixture was cooled to rt, water (500.0 mL) was added and its pH was adjusted to 2 with conc. HCl solution (50.0 mL). The precipitated white solid was filtered and washed with water. The solid was dried at 70–75 °C to a constant weight to give **9**. Yield = 56.0 g (90%); purity (HPLC): 99.9%; IR (KBr): 3220 (–COOH), 1682 (–CO–) cm^−1^; ^1^H NMR (CDCl_3_/TMS) δ 6.50 (d, 1H, –CH), 7.54 (t, 1H, ArH), 7.63 (d, 2H, –CH and ArH), 7.68–7.75 (m, 2H, ArH); ^13^C NMR (CDCl_3_/TMS) δ 172.62, 147.10, 140.03, 135.72, 134.15, 131.0, 129.80, 129.07, 129.03, 127.09, 125.71.

**Preparation of 3-(3-trifluoromethylphenyl)propionic acid (10):** Acid **9** (50.0 g, 0.231 mol) was dissolved in aqueous NaOH solution (500.0 mL) at rt. To the resulting clear solution, 5% Pd/C (1.0 g) was added and it was hydrogenated by bubbling H_2_ gas (1 bar) at 25–30 °C for 7 h. The reaction mixture was filtered through a pad of celite to separate the Pd/C catalyst. The pH of the filtrate layer was adjusted to 2 with conc. HCl solution (35.0 mL) at rt and the product was extracted with ethyl acetate (2 × 250.0 mL). The organic layer was dried over anhydrous Na_2_SO_4_ and evaporated under vacuum to get the product as colorless oil, which was recrystallized twice from *n*-hexane at 0 °C. Decanting the hexane layer from the crystalline solid affords the pure **10** (clear liquid at 25 °C). Yield = 47.3 g (90%); purity (GC–MS) = 99.45%; IR (neat): 3300–3400 (–COOH), 1712 (–CO–) cm^−1^; ^1^H NMR (CDCl_3_/TMS) δ 2.72 (t, 2H, –CH_2_), 3.03 (t, 2H, –CH_2_), 7.41 (d, 2H, ArH), 7.48 (s, 2H, ArH), 10.5 (broad s, 1H, –COOH); ^13^C NMR (CDCl_3_/TMS) δ 178.69, 140.92, 131.59, 130.97 (q), 128.89, 124.93, 123.9 (q), 123.23, 35.11, 30.17.

**Preparation of 3-(3-trifluoromethylphenyl)propionic acid methyl ester (11):** Acid **10** (50.0 g, 0.229 mol) was dissolved in methanol (200.0 mL) at rt and thionyl chloride (27.28 g, 0.229 mol) was added to this solution at rt. The clear solution was heated under refluxfor 4 h. After completion of the reaction, methanol was distilled off and the remaining reaction mixture was cooled to rt. Then, water (200.0 mL) was added and the pH of the mixture was adjusted to 7 with 5% aqueous sodium bicarbonate solution (150.0 mL). The product was extracted with ethyl acetate (2 × 250.0 mL) and the organic layer was dried over anhydrous Na_2_SO_4_. Then it was evaporated under vacuum to get the product as colorless oil. Yield = 52.0 g (97%); purity (GC) = 99.5%; IR (neat): absence of 3300–3400 (–COOH), 1740 (–CO–) cm^−1^; ^1^H NMR (CDCl_3_/TMS) δ 2.65 (t, *J* = 7.6, 2H, –CH_2_), 3.01 (t, *J* = 7.6, 2H, –CH_2_), 3.67 (s, 3H, –OCH_3_), 7.39 (d, *J* = 8.8, 2H, ArH), 7.46 (s, 2H, ArH); ^13^C NMR (CDCl_3_/TMS) δ 172.70, 141.31, 131.62, 130.72 (q), 128.80, 124.90, 123.99 (q), 123.09, 51.50, 35.17, 30.53.

**Preparation of 3-(3-trifluoromethylphenyl)propan-1-ol (12):** Compound **11** (50.0 g, 0.215 mol) was dissolved in THF (500.0 mL) and sodium borohydride (49.0 g, 1.295 mol) was added at rt. The resulting reaction solution was heated under reflux for 10 min. Methanol (500.0 mL) was added to this solution at 60–65 °C over a period of 4 h. After complete addition of methanol, the reaction solution was stirred for another 5 h to complete the reduction. After completion of the reaction, the solvent was distilled off and the remaining reaction mixture was cooled to rt, water (500.0 mL) was added and the pH was adjusted to 5 with conc. HCl solution (50.0 mL). The product was extracted with ethyl acetate (2 × 250.0 mL) and the organic layer was dried over anhydrous sodium sulfate and evaporated under vacuum to get the product as colorless oil. Yield = 43.5 g (95%); purity (GC) = 99.7%; IR (neat) 3350 (–OH), 2941, 2870, 1450, 1331, 1162, 1124, 1073 cm^−1^; ^1^H NMR (CDCl_3_/TMS) δ 1.90 (m, 2H, –CH_2_), 2.08 (s, 1H, –OH), 2.77 (t, *J* = 7.8, 2H, –CH_2_), 3.67 (t, *J* = 6.3, 2H, –CH_2_), 7.38 (d, *J* = 5.3, 2H, ArH), 7.45 (d, *J* = 5.3, 2H, ArH); ^13^C NMR (CDCl_3_/TMS) δ 142.64, 131.75, 130.54 (q), 128.67, 125.50, 124.99, 124.96, 122.8, 122.66, 122.63, 61.69, 33.80, 31.71.

**Preparation of 1-(3-bromopropyl)-3-trifluoromethylbenzene (5):** Compound **12** (50.0 g, 0.244 mol) was added to 48% aqueous HBr solution (400.0 mL) at rt. The reaction mixture was heated to 85–90 °C and stirred for 15 h. After completion of the reaction, the mixture was cooled to rt and water (250.0 mL) was added. The product was extracted with *n*-hexane (2 × 250.0 mL) and the organic layer was dried over anhydrous sodium sulfate and evaporated under vacuum to get the crude product, which was passed through a silica gel plug in *n*-hexane to afford the pure product as colorless oil. Yield = 53.6 g (82%); purity (GC) = 99.0%; IR (neat): absence of 3350 (–OH), 1451, 1330, 1164, 1125, 1094, 799, 702 cm^−1^; ^1^H NMR (CDCl_3_/TMS) δ 2.19 (quintet, 2H, –CH_2_), 2.86 (t, *J* = 7.3, 2H, –CH_2_), 3.41 (t, *J* = 6.5, 2H, –CH_2_) and 7.39–7.49 (m, 4H, ArH); ^13^C NMR (CDCl_3_/TMS) δ 141.36, 131.87, 130.67 (q, CF_3_), 128.83, 125.07, 123.03, 33.70, 33.67, 32.52.

**Preparation of 1-(3-iodopropyl)-3-trifluoromethylbenzene (6):** Compound **12** (50.0 g, 0.244 mol) was dissolved in CH_2_Cl_2_ (150.0 mL) and imidazole (2.0 g, 0.029 mol) and triphenylphosphine (70.64 g, 0.269 mol) were added at rt. To the resulting pale yellow clear solution was added iodine (62.0 g, 0.244 mol) in portions at less than 35 °C over a period of 1 h. The reaction mixture was stirred for 1 h and quenched with saturated sodium thiosulfate solution (2 × 100.0 mL) and water (250.0 mL). The product was extracted with *n*-hexane (2 × 250.0 mL) and the organic layer was dried over anhydrous sodium sulfate and evaporated under vacuum to get the product as colorless oil. Yield = 65.3 g (85%); purity (GC) = 99.45%; IR (neat): absence of 3100–3400 (–OH), 1450, 1330, 1164, 1126, 1074, 797, 702 cm^−1^; ^1^H NMR (CDCl_3_/TMS) δ 2.14 (quintet, 2H, –CH_2_), 2.80 (t, *J* = 7.3, 2H, –CH_2_), 3.41 (t, *J* = 6.6, 2H, –CH_2_), 7.39–7.49 (m, 4H, ArH); ^13^C NMR (CDCl_3_/TMS) δ 141.34, 132.01, 131.02 (q, CF_3_), 128.97, 125.53, 123.26, 36.02, 34.55, 5.72.

**Preparation of cinacalcet hydrochloride (1):** Compound **7** (3.0 g, 0.0065 mol) and MTBE (15.0 mL) were stirred for 15 min to become a clear solution. Conc. HCl solution (3.2 mL, 0.0129 mol) was added drop wise and stirred for 15 min at rt. The material was filtered, washed with MTBE and recrystallized from acetonitrile and water (1:2 ratio) at 65 °C for 2 h. The solid was dried at 50–55 °C to a constant weight to give pure hydrochloride salt of **1**. Yield = 2.1 g (91%); chiral purity (HPLC) = 99.95%; mp 174.6–176.8 °C; IR (KBr): 3427 (broad, –NH–), 2951, 2797, 2750, 2712, 1587, 1450, 1327, 1165, 1128, 1072, 798, 775 cm^−1^; ^1^H NMR (DMSO-*d*_6_/TMS) δ 1.67 (d, *J* = 6.6, 3H, –CH_3_), 1.99 (quintet, 2H, –CH_2_), 2.70 (m, 2H, –CH_2_), 2.93 (m, 2H, –CH_2_), 5.30 (q, 1H, –CH), 7.46–7.61 (m, 7H, ArH), 7.95–8.03 (m, 3H, ArH), 8.23 (d, *J* = 8.0, 1H, ArH), 9.36 (s, 1H, –NH) and 10.04 (s, 1H, HCl); ^13^C NMR (DMSO-*d*_6_/TMS) δ 142.61, 134.48, 133.70, 132.80, 130.64, 129.74, 129.58,129.28, 127.29, 126.54, 125.90, 125.08, 124.67, 123.16, 122.98, 52.37, 45.04, 31.84, 27.44, 20.30; MS *m*/*z:* 358 [M + 1]^+^.

## Supporting Information

File 1 ^1^H NMR, ^13^C NMR and ESI–MS spectra of compounds **1**, **4**, **5**, **6** and **7**.
