# Supplementary material for: A novel asymmetric synthesis of cinacalcet hydrochloride
Source: Beilstein J Org Chem. 2012 Aug 24;8:1366–73. doi: 10.3762/bjoc.8.158 (PMC3458763; doi:10.3762/bjoc.8.158)
Supplement: File 1 — 1H NMR, 13C NMR and ESI–MS spectra of compounds 1, 4, 5, 6 and 7. [file Beilstein_J_Org_Chem-08-1366-s001.pdf]

Supporting Information

for

A novel asymmetric synthesis of cinacalcet hydrochloride

Veera R. Arava[1]*, Laxminarasimhulu Gorentla[1] and Pramod K. Dubey[2]

Address: [1]R&D Laboratory, Suven Life Sciences Ltd., Hyderabad, India and [2]Department of Chemistry, J. N. T. University, Hyderabad, India

Email: Veera Reddy Arava* - reddyvenis@rediffmail.com; Laxminarasimhulu Gorentla - laxman164@rediffmail.com; Pramod Kumar Dubey - pramoddubey7@gmail.com

*Corresponding author

^{1}H NMR, ^{13}C NMR and ESI–MS spectra of compounds 1, 4, 5, 6 and 7

Table of contents

^{1}H and ^{13}C NMR spectra of compound **4** S3

ESIMS of **4** and ^{1}H NMR spectrum of **5** S4

^{13}C NMR of **5** and ^{1}H NMR spectrum of compound **6** S5

^{13}C NMR of **6** and ^{1}H NMR spectrum of compound **7** S6

^{13}C NMR and ESIMS spectra of compound **7** S7

^{1}H and ^{13}C NMR spectra of compound **1** S8

ESI–MS spectrum of **1** S9

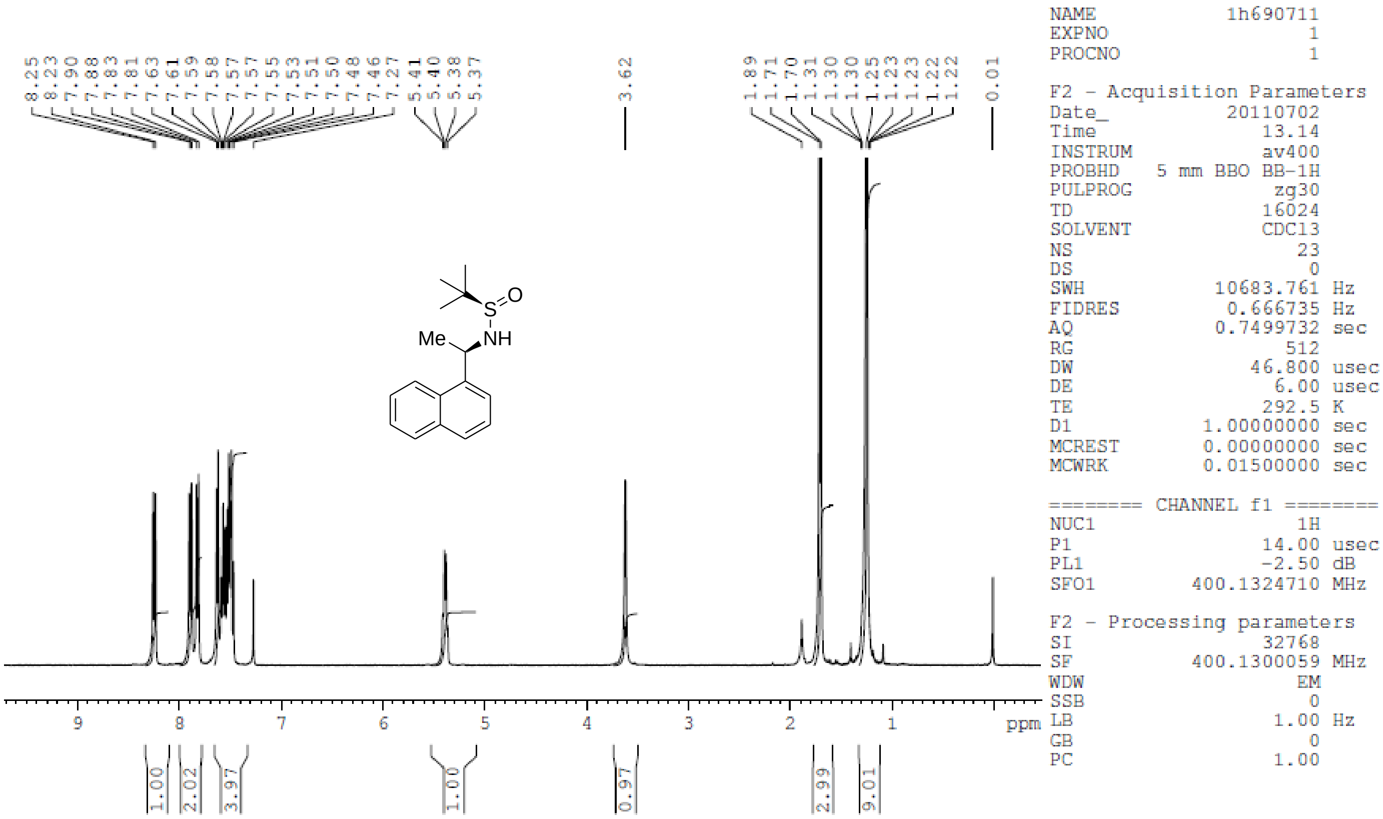

^{1}H NMR spectrum of compound **4**

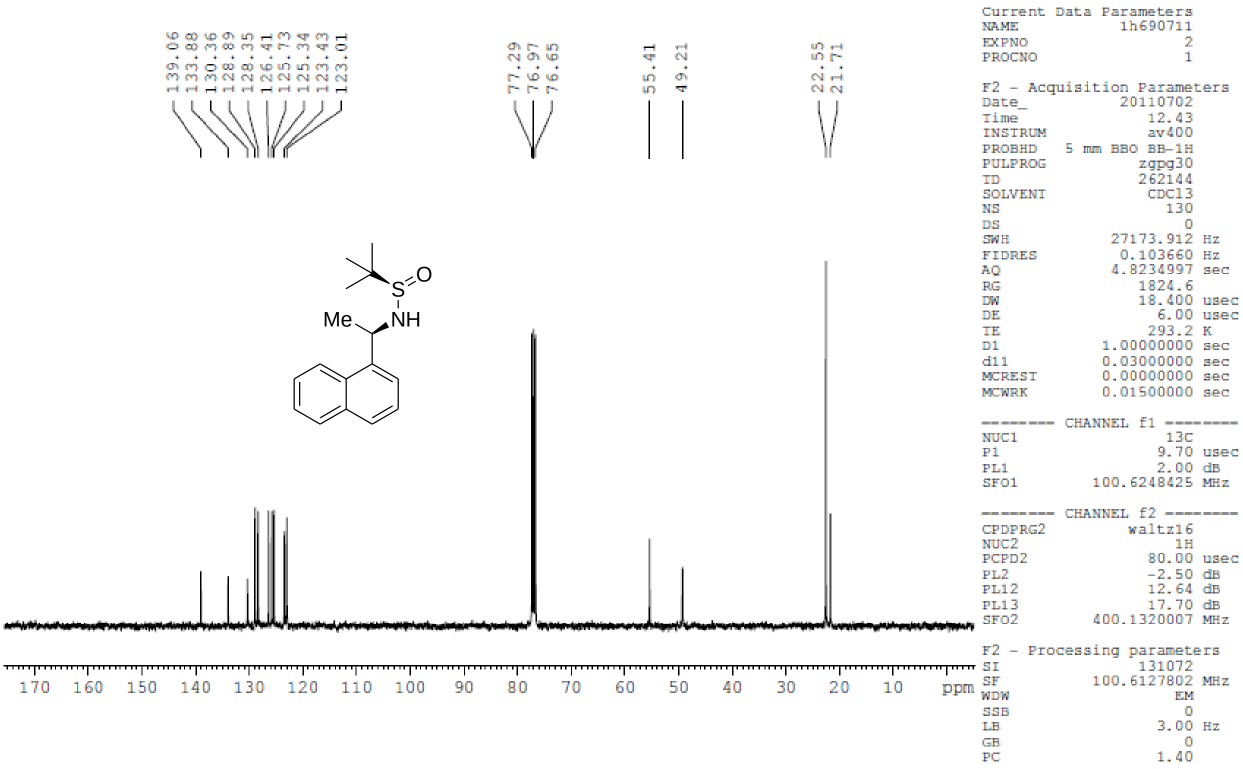

^{13}C NMR spectrum of compound **4**

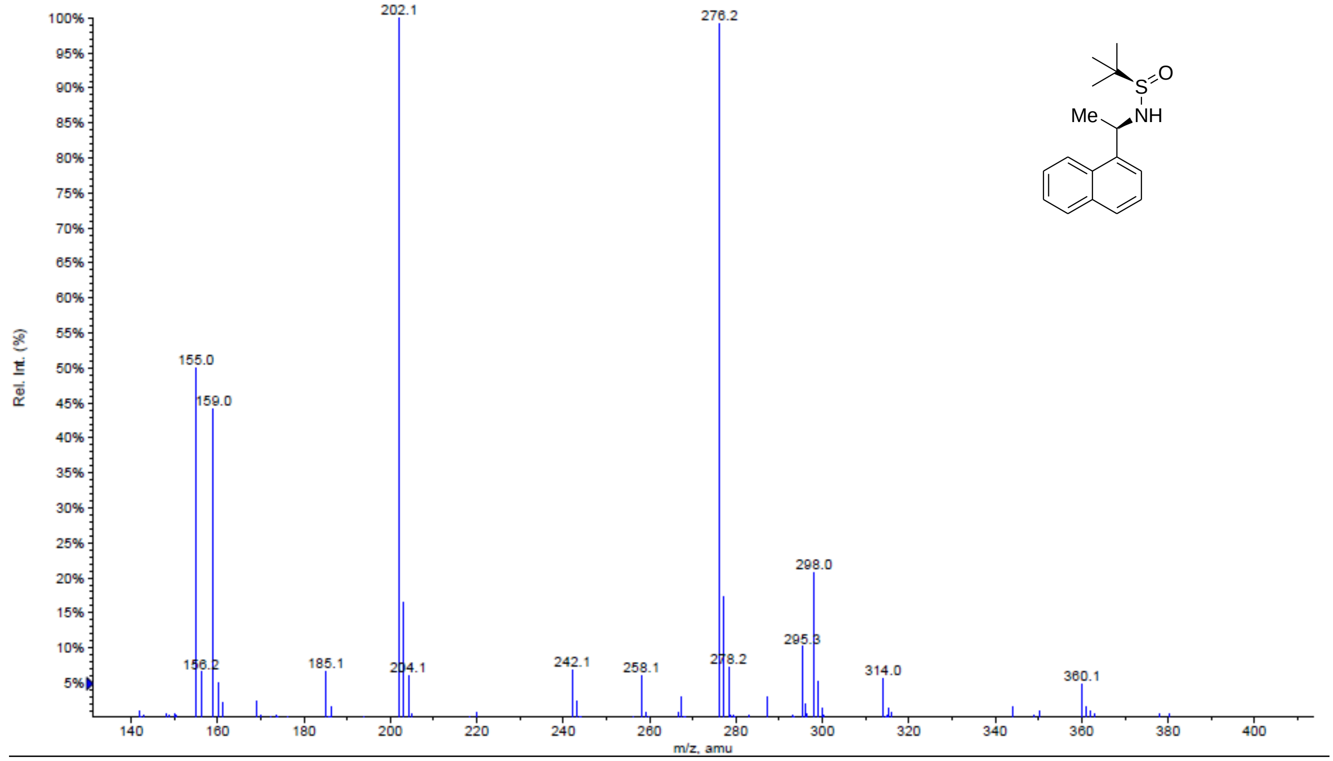

ESIMS spectrum of compound **4**

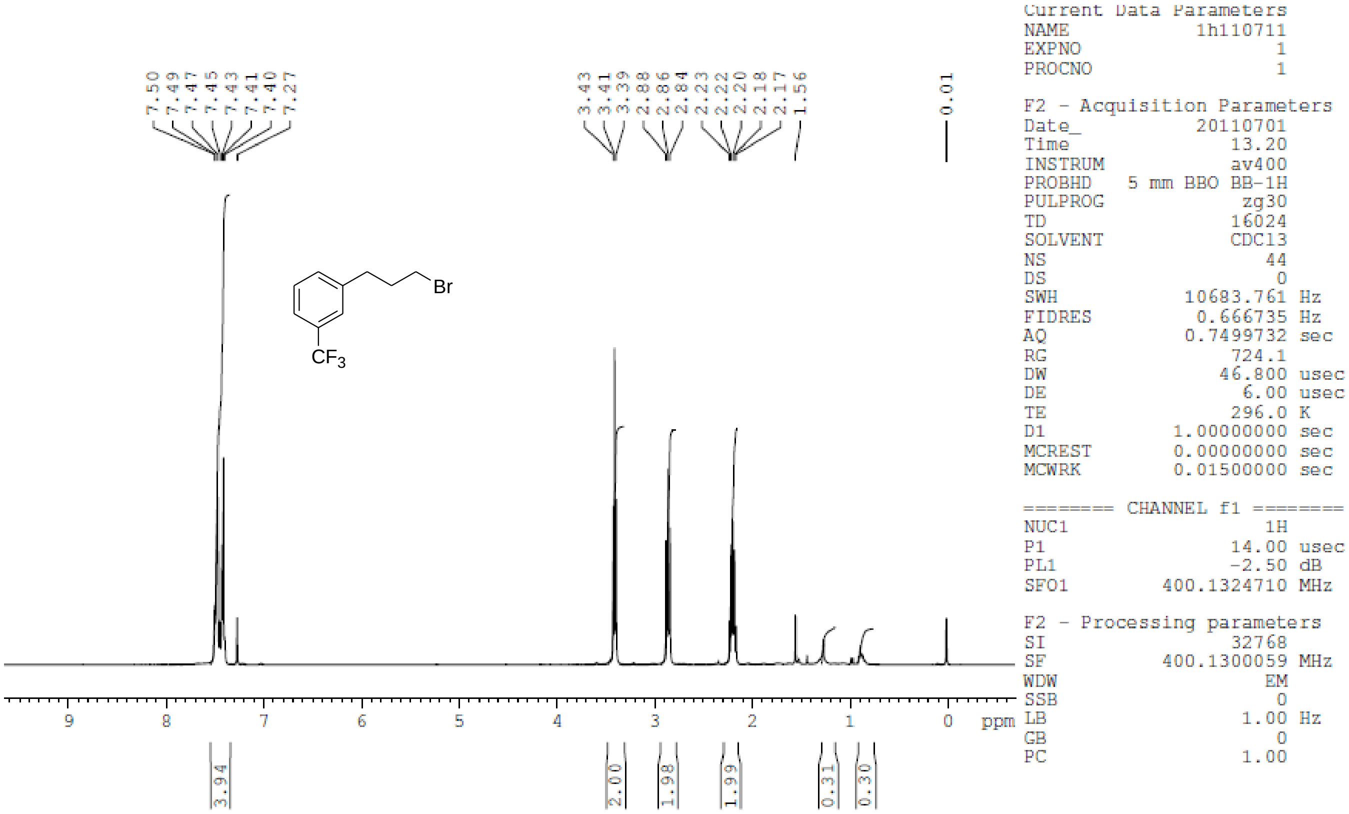

^{1}H NMR spectrum of compound **5**

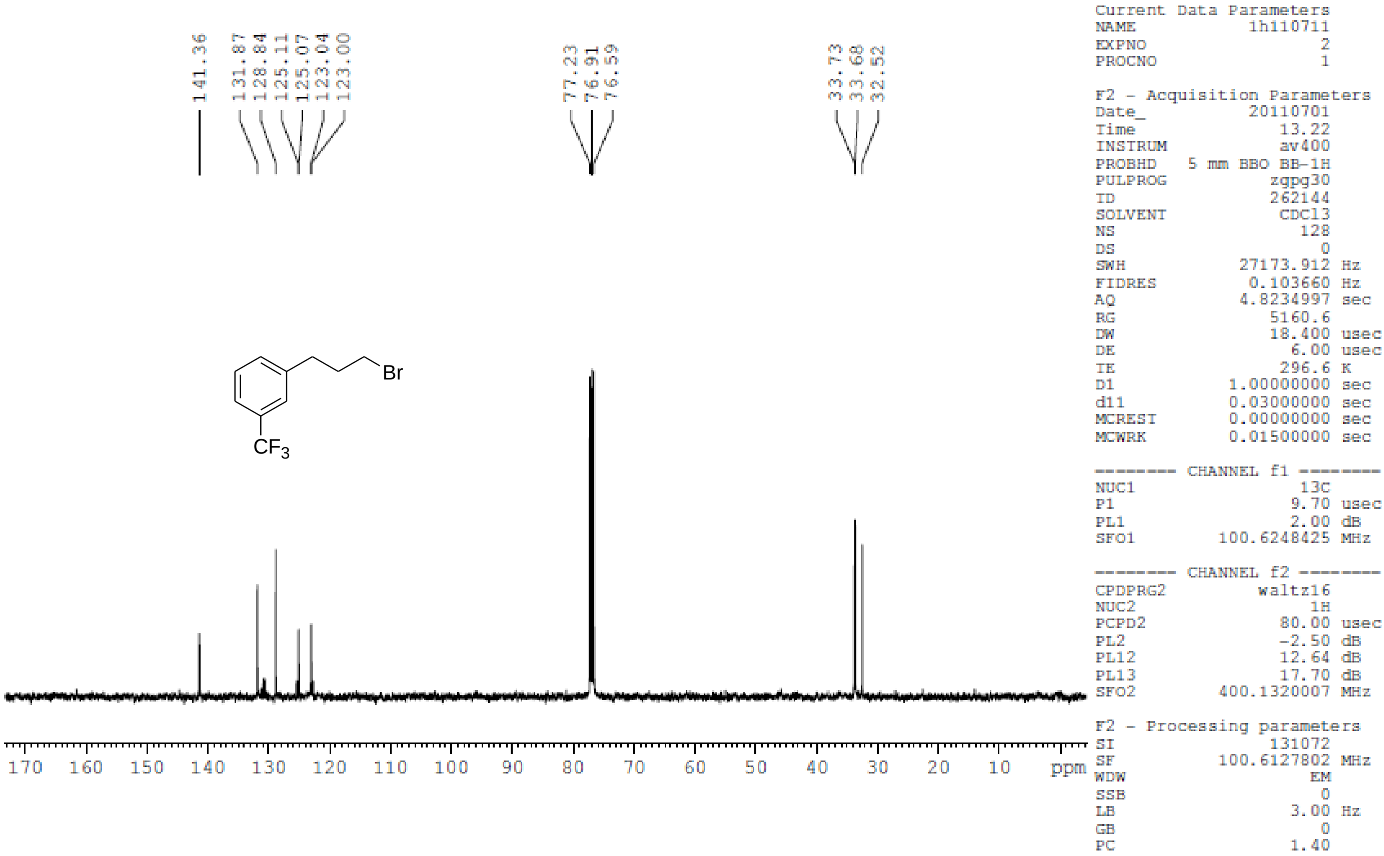

^{13}C NMR spectrum of compound **5**

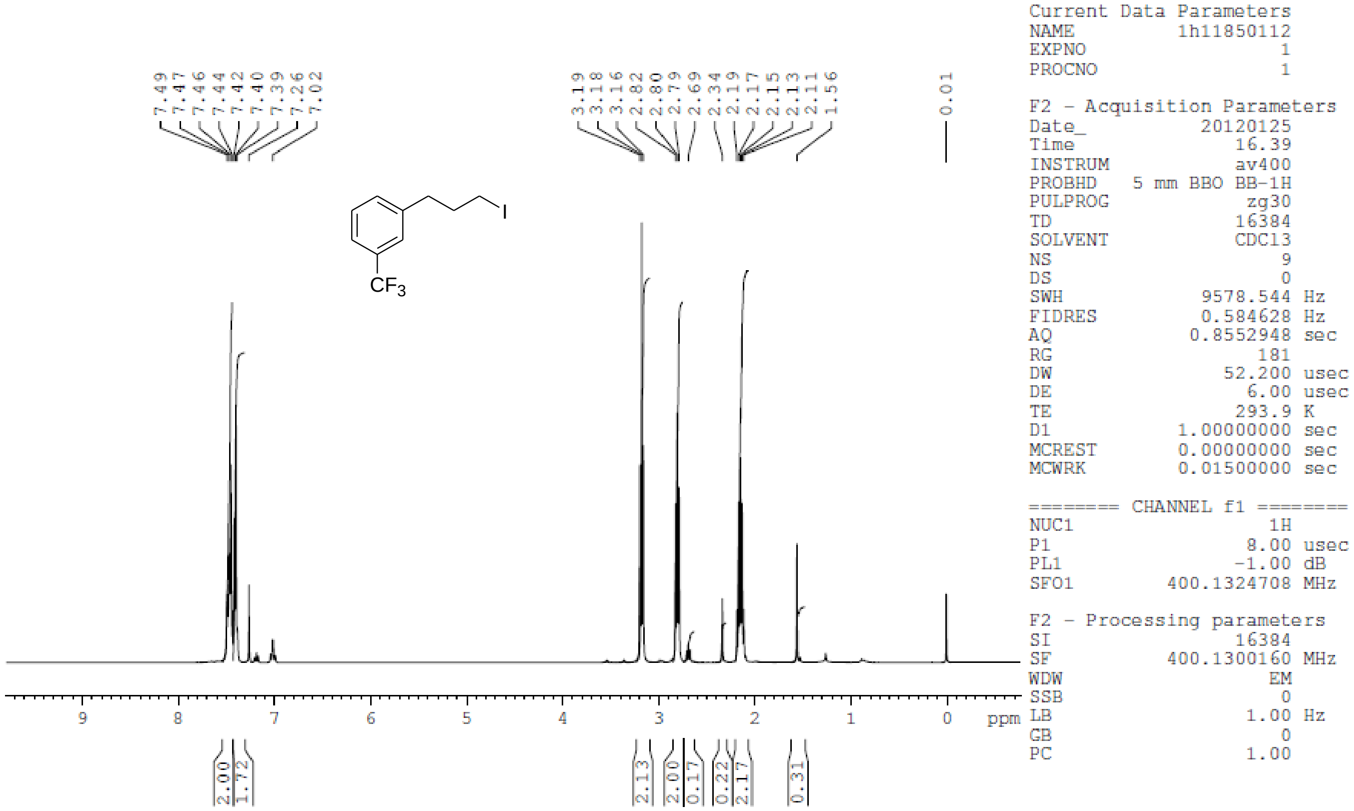

^{1}H NMR spectrum of compound **6**

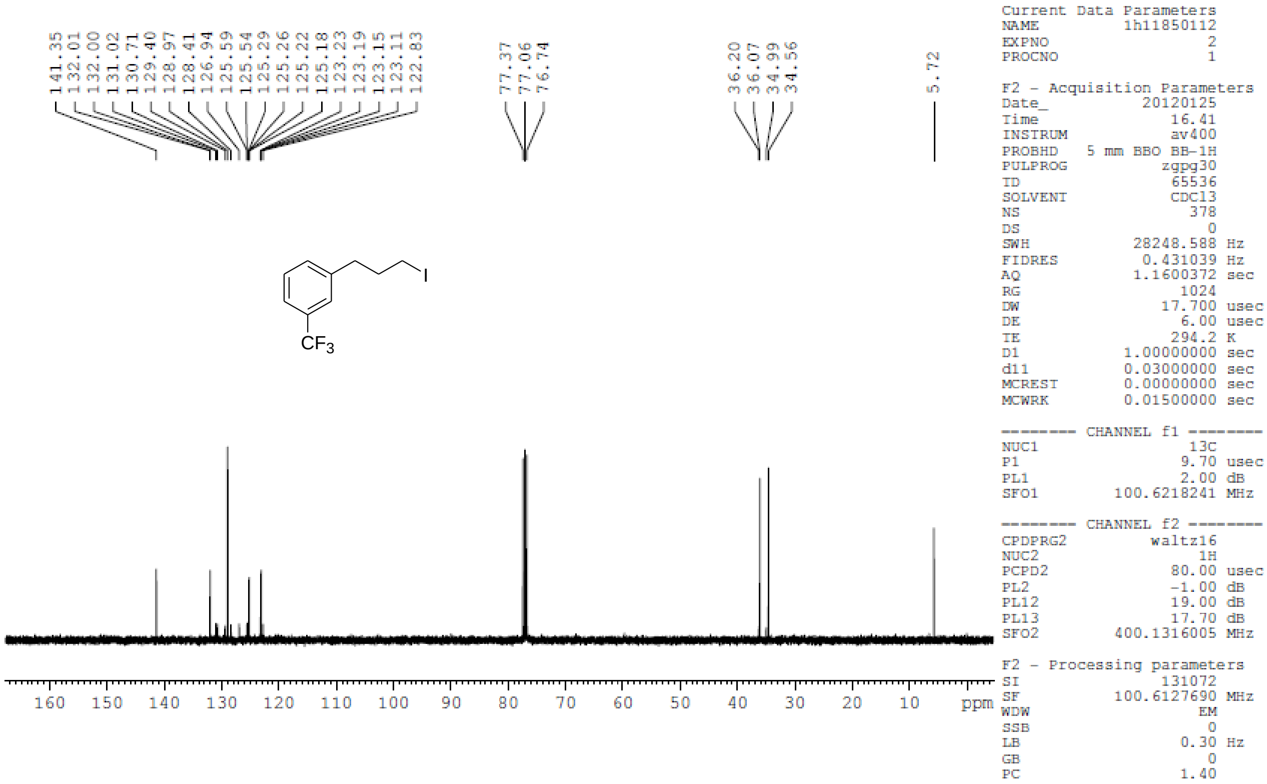

^{13}C NMR spectrum of compound **6**

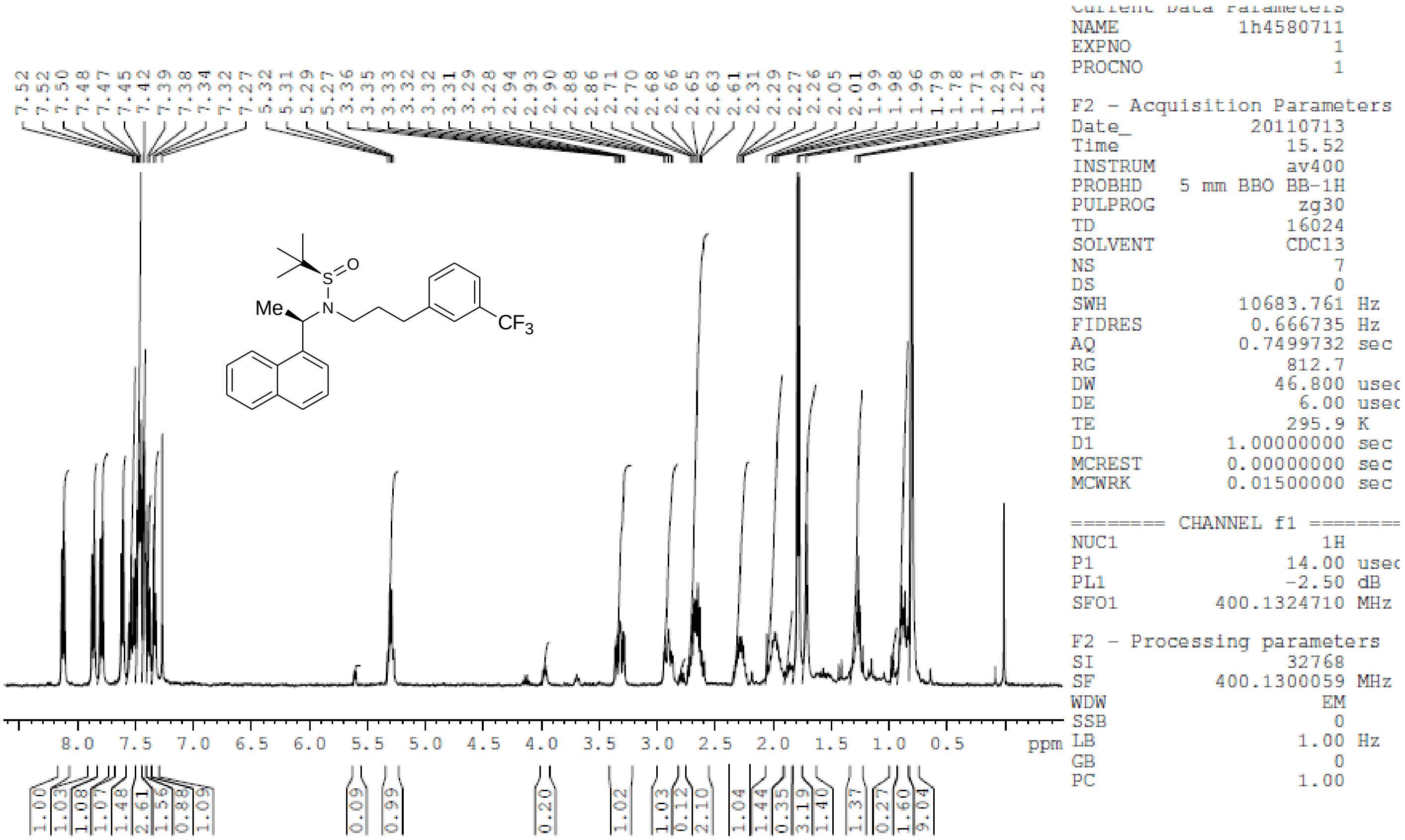

^{1}H NMR spectrum of compound **7**

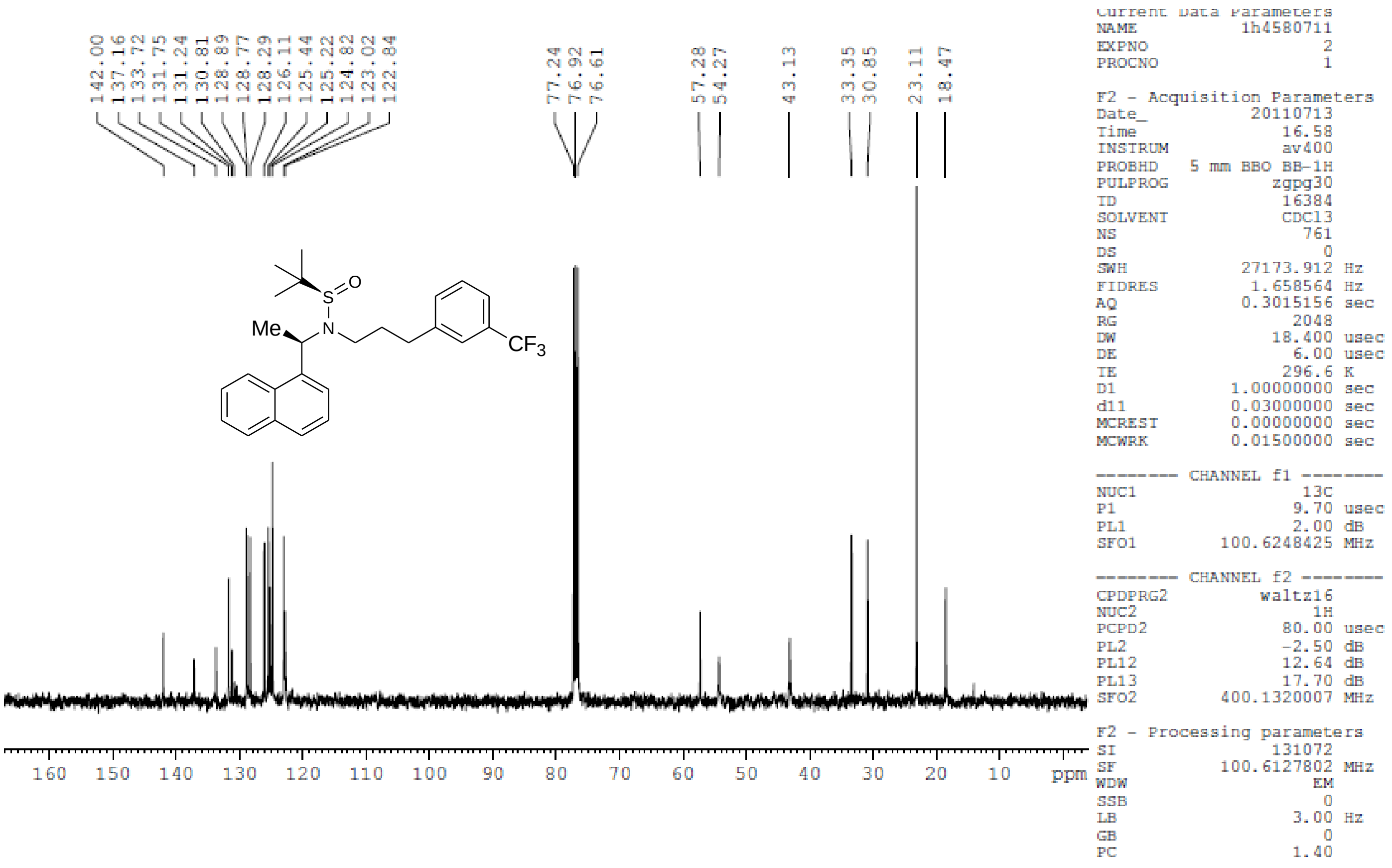

^{13}C NMR spectrum of compound **7**

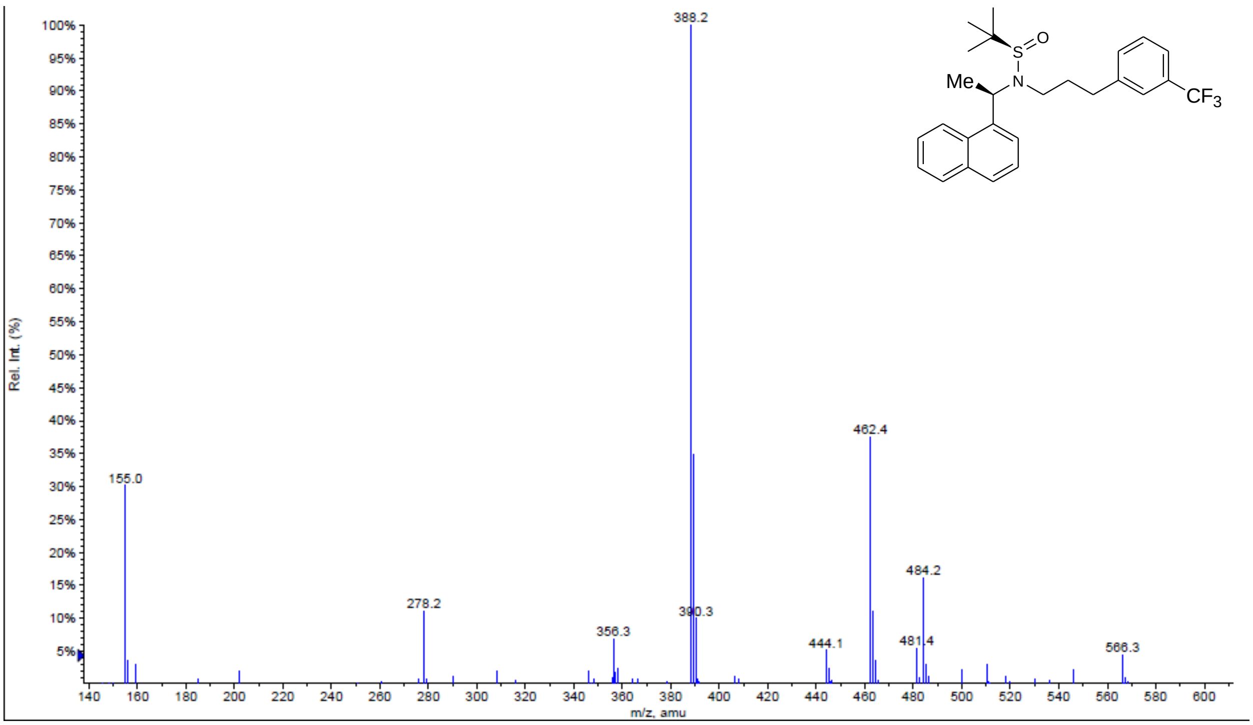

ESIMS spectrum of compound **7**

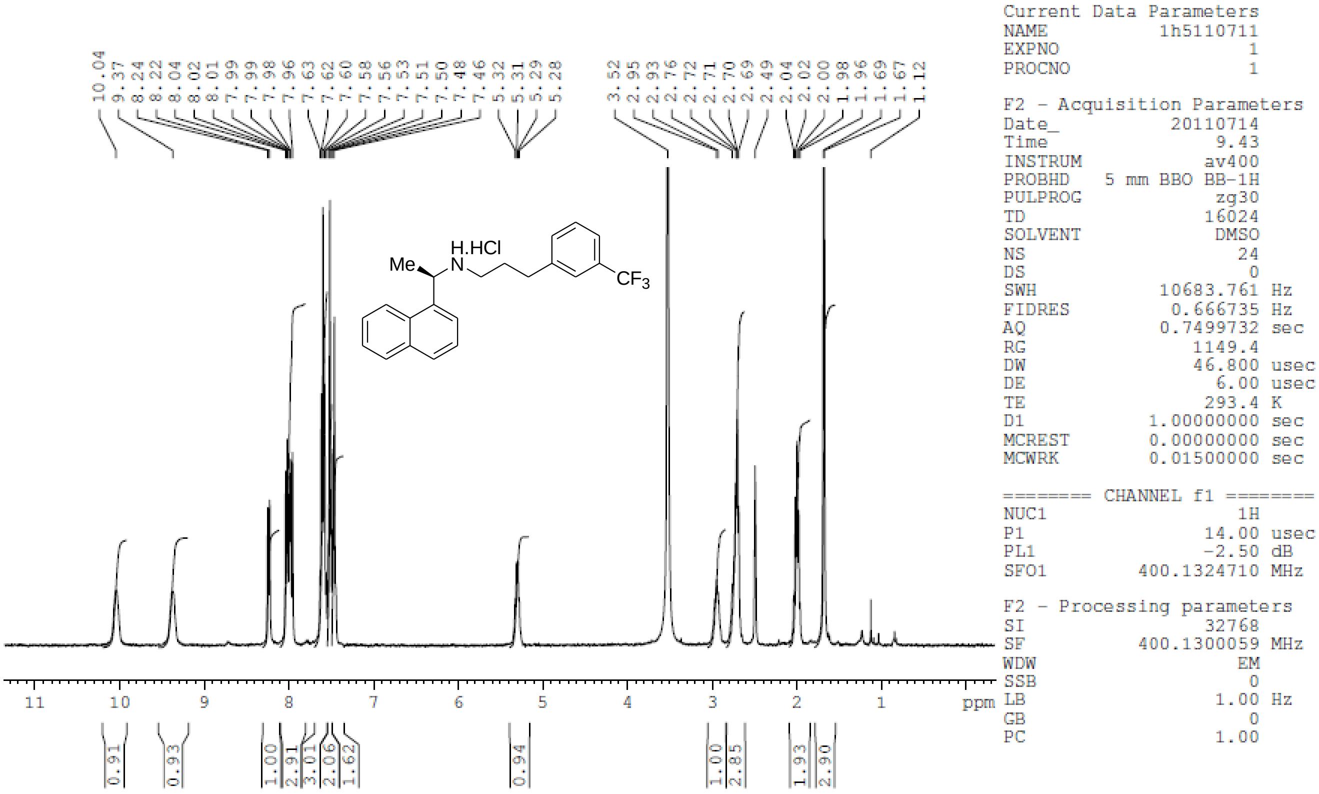

^{1}H NMR spectrum of compound **1**

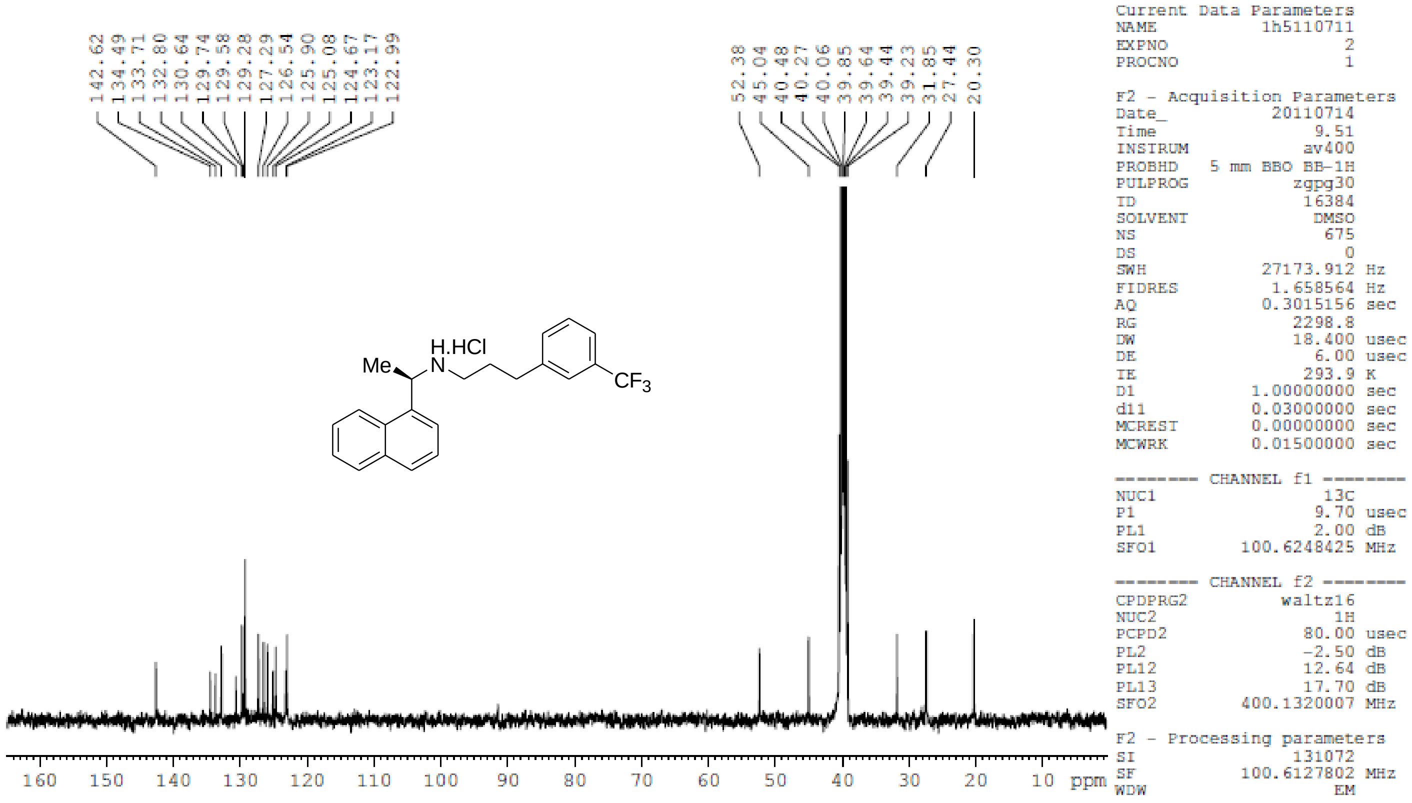

^{13}C NMR spectrum of compound **1**

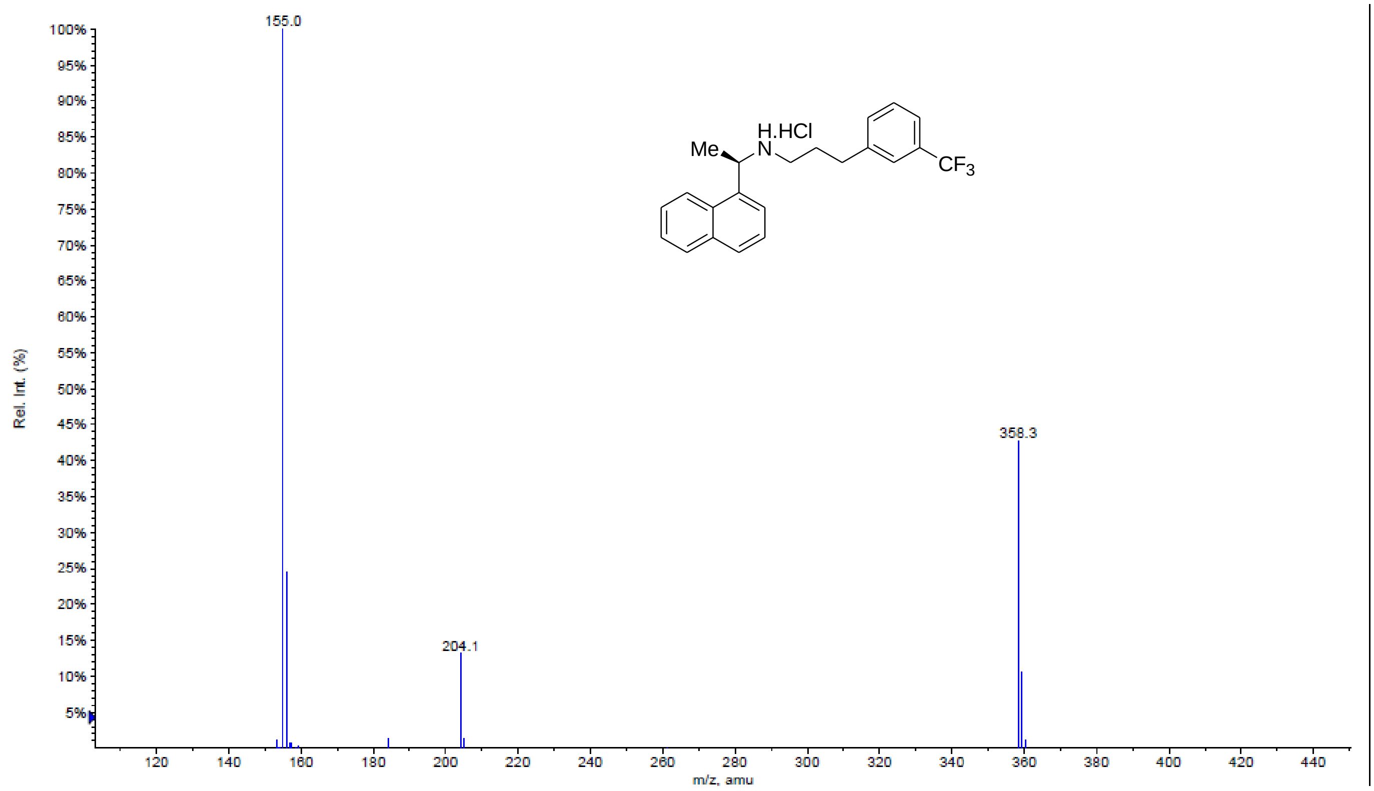

ESIMS spectrum of compound **1**
